# Marketing by online tobacco retailers: An observational cross-sectional study

**DOI:** 10.18332/tid/220983

**Published:** 2026-06-19

**Authors:** Dorie E. Apollonio, Cathi E. Dennehy, Candy Tsourounis, Tanner Wakefield

**Affiliations:** 1 School of Pharmacy, University of California, San Francisco, United States; 2 School of Medicine, University of California, San Francisco, United States

**Keywords:** tobacco, marketing, cannabis, marijuana

## Abstract

**INTRODUCTION:**

Understanding retailer advertising has been identified as critical for developing effective policies to reduce tobacco product use, as these forms of advertising are among the tobacco industry’s largest expenditures. Online retailer marketing has grown rapidly, and in this study, we sought to describe the marketing practices of these retailers.

**METHODS:**

Between March and July 2022, we conducted keyword searches (e.g. ‘buy e-cigs’, ‘buy vapes’) with the Brave search engine, embedded in the Brave browser, to identify online tobacco retailers, using the inclusion criteria: English-language websites of online retailers selling e-cigarette products that allowed online ordering and the sale of products to customers in the United States. Measures included name, address, and landing page characteristics, including products, brands, product types, seasonal specials, social media links, age gating, and whether the retailer sold non-tobacco products. Results are reported descriptively.

**RESULTS:**

We identified 97 unique online tobacco retailers. Of these, 58 (60%) had set a restriction on browsing based on age. E-cigarettes, both disposable and reusable, were the most available products, followed by liquid nicotine (‘vape juice’). Thirty-seven percent of online tobacco retailers sold cannabis products, and 38% of retailer websites listed other types of products for sale (e.g. bongs, dab rigs, cannabis apparel, psilocybin chocolates).

**CONCLUSIONS:**

Our findings indicated that online tobacco retailers heavily marketed flavored products, and a majority sold derived cannabis products. Future research should continue to investigate whether this marketing conflicts with stated federal regulatory goals, and whether the U.S. Food and Drug Administration should expand enforcement of existing regulations on tobacco and derived cannabis products.

## INTRODUCTION

In 2018, the U.S. Food and Drug Administration (FDA) announced that e-cigarette packaging had become increasingly similar to candy and juice products that children could mistake for real food^1,2^. Understanding the advertising approach on retailer websites is recognized as critical to effective tobacco control^3^ because these kinds of advertising are among the tobacco industry’s largest expenditures. Retailer website marketing has rapidly increased since the Master Settlement Agreement, an accord reached in November 1998 between the 46 Attorneys General and the four largest US tobacco companies that banned traditional advertising^4^. In 2021 (the most recent year of the U.S. Federal Trade Commission E-Cigarette Report), e-cigarette companies spent >$850 million on advertising and promotions; >$24 million of that total was directed to online marketing, and >$96 million to point-of-sale advertising^5^. Despite Master Settlement Agreement restrictions, tobacco product advertising is particularly effective at reaching young people in the US; by 2019, nearly 80% of youth aged 13–17 years had been exposed to e-cigarette advertisements, most commonly at point-of-sale (58%) and online (50%)^6^. Youth exposure to e-cigarette marketing increases the likelihood of tobacco use in the past 30 days, and of ever use^7^. Retail stores, including online stores, are the most common source of exposure^8^. The internet has been, and remains, a primary medium for marketing tobacco products^9^, yet there is limited recent information on the nature of marketing claims made by online retailers^3^.

The FDA regulates the marketing of tobacco retailers. This includes a requirement that covered tobacco products include the warning, ‘This product contains nicotine. Nicotine is an addictive chemical’^10^. The FDA will also notify retailers of violations of existing regulations – for example, selling tobacco products to minors – through a Warning Letter; however, previous studies have found that online retailers rarely remediate such violations^11,12^.

Limited enforcement of minimum age of sales policies contributes to increased use of tobacco products by young people^13^. This issue is particularly acute for online retailers. A 2014 study found that one-fifth of storefront (brick-and-mortar) retailers allowed in-person underage tobacco purchases^14^. In contrast, a 2015 study found that over three-quarters of online tobacco purchase attempts by minors were successful; delivery companies did not verify age, and 95% of orders were left at the door^15^. Online retailers typically ask visitors to self-report their age using ‘click-through’, in which users are asked to confirm that they are of legal age before proceeding to a product page, but companies do not verify the claim^3,16^. In addition, recent research has noted that tobacco retailers that sell electronic nicotine delivery systems (ENDS) are more likely to sell cannabis products due to the demographics of target populations and changes in tobacco and cannabis regulations^17,18^; cannabis use also increases the likelihood of tobacco product use^19^.

The aim of this study was to describe the marketing practices of online tobacco retailers. Understanding retailer advertising has been identified as critical in developing effective policies to reduce tobacco product use^3^.

## METHODS

We conducted an observational cross-sectional study of online tobacco retailers, using search strategies and methods validated in prior research^20^.

### Data source and collection

As a data collection strategy, we relied on methods validated in prior studies, in which online retailers were identified using searches beginning with ‘buy’ followed by different potential product names^21,22^, e.g. ‘buy ecigarettes’, ‘buy e cigs’, and ‘buy vapes’. In July–August 2022, we used lists of commonly used product names identified by the FDA and Truth Initiative to conduct multiple online searches for retailers^23,24^. Searches were conducted using the Brave search engine, embedded in the Brave browser, which blocks trackers and third-party cookies by default, ensuring each search returned unique results. A detailed list of search terms, search dates, and identified sites is provided in the Supplementary file. We identified online retailers from both their own sites and from links on aggregation sites such as ‘7 Best Online Vape Stores’, until our searches reached a duplication rate of ≥90%, indicating saturation. We applied the following inclusion criteria: English-language websites of online retailers selling e-cigarette products that allowed online ordering and the sale of products to US customers. We included retailers that also sold other products in addition to tobacco [most commonly, derived cannabis products such as cannabidiol (CBD) and derived psychoactive cannabis products such as delta-8^25^]. One author (TW) screened the resulting list of retailers for duplicates and generated the final sample with each unique retailer listed once.

### Coding and measures

We used REDCap, a secure, web-based software platform designed to collect and manage study data^17,18^ to aggregate and code preliminary data^26,27^. The instrument was developed based on past studies of online retailers^11^. All the authors pilot tested the instrument, reviewed it together by coding the first three websites identified, then retested it by double-coding ten randomly selected retailer sites, at which point the group reached consensus. After this point, one author coded each retailer site individually, and the investigators met and discussed any sites where claims appeared unclear, until reaching consensus.

Each retailer was coded for: retailer name, website address, and contact information if available. Within the US, retailer geographical locations were classified using Census Regions and Divisions. In addition, we abstracted information regarding the website and products. This included coding the characteristics of the landing page: number of products and brands listed, types of products available, seasonal specials (if any), links to social media, the presence or absence of age-gating and its characteristics (e.g. age allowed for entry, verification type), and whether the retailer sold other types of products such as those marketed and labeled as cannabis or herbal supplements.

The FDA requires that covered tobacco products include the warning, ‘This product contains nicotine. Nicotine is an addictive chemical’^10^. We indicated whether this warning was present. We also coded the types (e.g. disposable e-cigarette, reusable e-cigarette, chewing tobacco), characteristics (e.g. flavored), and prices (listed in US$ for all retailers) of the first three products listed for sale. We identified the first three products listed for sale in one of two ways: on a static webpage, we coded products in order as listed left to right and top to bottom on the landing page; on a webpage that contained a banner or carousel at the top that highlighted different products by scrolling through them over time, we coded the first three products in order of appearance. For each retailer, we noted the date we reviewed the information and saved screenshots of each landing page and any age-gating (if any). For each product, we saved screenshots of the product page. Finally, we reviewed the FDA’s online database of Warning Letters to determine whether retailers identified through searches and included in the sample had previously been notified of marketing violations.

### Statistical analysis

We used Stata v17 for all quantitative analyses. We calculated descriptive statistics for different types of marketing claims and product types. We also assessed marketing claims made by online retailers in each category^28-33^. We assessed the frequency and types of age verification on online retailer sites, anticipating that most retailers used click-through verification or no verification^3,16^.

### Ethical approval

The research was conducted using publicly accessible data without special permission or application and, as a result, was not considered to involve human subjects (UCSF IRB #10-01262).

## RESULTS

Our final sample of unique online tobacco retailers, after exclusions (e.g. duplicates, no sales to US customers), totaled 97 websites. Of these 97 websites, 13 (13%) provided a physical address; 3 retailers listed locations outside the US, 2 in Canada, and 1 in the United Arab Emirates (UAE). Of the remaining 10 retailers, 5 (50%) were in the South, 3 (30%) in the West, 1 (10%) in the Northeast, and 1 (10%) in the Midwest. Although all the retailers in our sample sold products to US consumers, the other 84 retailers did not provide location information, making it unclear whether they were operating within or outside the US. Retailers primarily provided contact information by listing an email address (91 sites, 94% of the sample).

### Extent of age restrictions on browsing by minors

Of the retailers in this sample, 58 (60%) had implemented an age-based browsing restriction; the remaining 39 (40%) did not attempt to restrict access by minors. Among the retailers that indicated there was an age restriction on browsing, the majority (45; 46% of the sample and 78% of those with age restrictions) were consistent with US laws that restricted the sale of tobacco products to people aged ≥21 years as of December 2019^34^ (prior to this date, minimum age of sale policies ranged from 18 to 21 years, depending on the state). The remaining 13 indicated that individuals aged ≥18 years browse (5 retailers), that visitors must be of ‘legal age’ (6 retailers), or (incorrectly) that eligibility to purchase was dependent on state law (2 retailers). Results are provided in Table 1.

**Table 1:** Online retailer website characteristics, observational cross-sectional study, USA, 2022 (N=97)

*Characteristics*	*n*	*%*
**No age restriction on browsing**	39	40
**Age restrictions on browsing**	58	60
≥21 years	45	46
≥18 years	5	5
‘Legal age’	6	6
Claimed age restriction depends on state law	2	2
**Required warning statement**		
Posted	59	61
Not posted	38	39
**FDA reported a previous marketing violation**	67	69
No required marketing authorization	58	60
Missing nicotine warning on e-liquids	9	9
Manufacturing in an unregistered establishment	2	2
Other	2	2
Types of products sold*		
Disposable e-cigarettes	72	74
Reusable e-cigarettes	69	71
E-cigarette liquids (‘vape juice’)	64	66
Accessories	62	64
Kits containing multiple products	56	58
Cannabis products	36	37
E-cigars	9	9
Chewing tobacco	1	1
Combustible cigarettes	0	0

FDA: U.S. Food and Drug Administration.

* Most available of multiple product types.

One online tobacco retailer in the sample suggested that consumers have packages delivered to workplaces or to drop-off locations, such as hardware stores, to secure an adult signature. The retailer EightVape stated on its shipping page in October 2023 (as captured by screenshot) that, ‘An adult (21+ years of age) is required to provide a valid government-issued ID upon delivery and must sign for the package. Think about delivering to your work or where someone is present to receive your order’^35^.

### Inclusion of legal warning and history of prior violations

As described above, the FDA imposes restrictions on the sales of tobacco products, including the requirement that tobacco products include a warning statement. Among the retailers in this sample, 38 (39%) did not include the required warning on their website landing pages.

Among the 97 online retailers in the sample, 67 (69%) had been advised of a prior violation. Most of these violations were for selling products without the required marketing authorization (58 retailers; 87%). The remainder were for e-liquid products that failed to include the required nicotine warning statement (9 retailers; 13%); in addition, 2 retailers had received additional warnings for manufacturing products in an unregistered establishment, 1 for failing to pay previously assessed fees, and 1 for marketing tobacco products using ‘cartoon unicorns’.

### Types of products available on retailer websites

Retailers in the sample sold multiple product types, as highlighted in Table 1. E-cigarettes, both disposable (74%) and reusable (71%), were the most available products, followed by liquid nicotine (‘vape juice’). In addition, 37% of online tobacco retailers sold cannabis products (CBD or derived psychoactive cannabis products such as delta-8^25^). In addition, 38% of retailer websites listed other types of products for sale, including bongs, dab rigs, cannabis apparel, psilocybin chocolates, loose-leaf tobacco, e-hookah, nicotine gum, fidget spinners, masks, hand sanitizer, lanyards, candles, and cleaning supplies. Only 16 retailers (17%) represented a single producer; the remainder offered products from multiple brands. The number of products visible on the website landing page ranged from 0 (3 retailers) to 82 (1 retailer). None of the retailers in the sample sold combustible cigarettes.

## DISCUSSION

This study suggests a significant gap between federal regulatory goals and the actual practices of online vendors^2,4^. We found that over two-thirds of retailers, identified through a simple internet search designed to mimic consumer behavior, had a history of prior FDA violations, primarily for selling products without required marketing authorization^5^. Furthermore, over a third of retailers failed to display the mandatory nicotine warning on their landing pages^5,6^. This high rate of non-compliance, combined with the fact that many retailers do not provide a physical address, complicates enforcement efforts^5,7^, and suggests that current enforcement mechanisms do not deter online retailers from violating federal policies. Although the FDA continues enforcement actions against unauthorized tobacco products, its focus has been on products that have not secured marketing authorization^36^.

The study reinforces concerns regarding youth access to tobacco products via online retailers identified in the past^12^. Although slightly more than half of retailers placed some form of age restriction on browsing, over one-third did not^6,8^. Even among those that imposed browsing restrictions, many used ‘click-through’ verification or cited an incorrect minimum age of sale (such as 18 years) that was inconsistent with the federal ‘Tobacco 21’ law^8^. Earlier studies found that online age verification is often easily bypassed, with some retailers suggesting delivery to workplaces to circumvent adult signature requirements at home^9,11^.

The findings also identified a convergence of tobacco and cannabis marketing. Over one-third of online tobacco retailers also sold CBD (cannabidiol) or derived psychoactive cannabis products like delta-8. Some retailers had further diversified into ‘lifestyle’ and other psychoactive products, including psilocybin chocolates and cannabis-related apparel^1,12^. These results suggest that consumers seeking tobacco products are systematically introduced to other substances by retailers, potentially for the first time^14^, through marketing of disposable e-cigarettes and cross-promotion of tobacco and derived psychoactive cannabis products. Our findings, coupled with prior research that also showed widespread availability of flavored tobacco products and cross-marketing of cannabis products, suggest that marketing by online retailers could lead young people to perceive tobacco and cannabis products as safe to consume.

### Limitations

The study was a cross-sectional analysis conducted at a single point in time. By their nature, observational studies cannot establish causal relationships. Because we did not conduct actual purchases, we could not assess whether age verification occurred at the point of sale or delivery. It was not possible to assess whether the search terms were comprehensive; as a result, we may have reviewed a more limited sample of online retailers than intended, potentially limiting generalizability. Future research should investigate whether these marketing practices remain consistent over time and the effectiveness of requiring adult signatures for age-restricted shipments.

## CONCLUSIONS

Tobacco retailers who sell products online do not provide clear information about their locations, and retailer communication is limited to email. This study of retailers also shows a strong predisposition to ignore existing regulations: most online tobacco product retailers identified through common search terms had previously been notified by the U.S. Food and Drug Administration of marketing violations. These retailers also failed to consistently restrict browsing to consumers who met the minimum purchase age or to include required warning statements about the risks of tobacco products. Future research should continue to investigate whether this marketing conflicts with stated federal regulatory goals, and whether the U.S. Food and Drug Administration should expand enforcement of existing regulations on tobacco and derived cannabis products.

## Supplementary Material



## Data Availability

The data supporting this research can be found in the Supplementary file.
