# Supplementary material for: Marketing by online tobacco retailers: An observational cross-sectional study
Source: Tob Induc Dis. 2026 Jun 19;24:10.18332/tid/220983. doi: 10.18332/tid/220983 (PMC13280932; doi:10.18332/tid/220983)
Supplement: Supplementary file 1 [file TID-24-93-s1.pdf]

Supplementary file Table 1

Date of search (xx/xx/xxxx)	Search term used ("buy____")	Link for each website of first google page
**7/20/2022**	vape	https://www.elementvape.com/
**7/20/2022**	vape	https://vaping.com/
**7/20/2022**	vape	https://www.vaporfi.com/
**7/20/2022**	vape	https://www.directvapor.com/
**7/20/2022**	vape	https://vaping360.com/best-beginner-e-cigs-vapes/
**7/20/2022**	vape	https://vapingvibe.com/best-nicotine-free-vape-products/
**7/20/2022**	vape	https://vapesourcing.com/
**7/20/2022**	vape	https://versedvaper.com/best-online-vape-stores/
**7/20/2022**	vape	https://www.eightvape.com/?sscid=71k6_kb88t
**7/20/2022**	vape	https://www.centralvapors.com/?sscid=71k6_kb89l
**7/20/2022**	vape	https://myvpro.com/?sscid=71k6_kb89z
**7/20/2022**	vape	https://vapordna.com/?sscid=71k6_kb8au
**7/20/2022**	vape	https://www.directvapor.com/?sscid=71k6_kb8be
**7/20/2022**	vape	https://www.ejuice.deals/?sscid=71k6_kb8cn&utm_source=ShareASale
**7/20/2022**	vape	https://www.ejuices.com/?utm_source=27001&utm_content=1077519_1700915&sscid=71k6_kb8e2
**7/20/2022**	vape	https://vapejuicedepot.com/?sscid=71k6_kb8ew&
**7/20/2022**	vape	https://mipod.com/?sscid=71k6_kb8g9&utm_source=ShareASale&utm_medium=Affiliate&utm_campaign=1700915
**7/20/2022**	vape	https://breazy.com/
**7/20/2022**	vape	https://blazedvapes.com/
**7/20/2022**	vaporizer	https://www.planetofthevapes.com/
**7/20/2022**	vaporizer	https://www.namastevaporizers.com/
**7/20/2022**	vaporizer	https://www.gearpatrol.com/home/g38569746/best-weed-vaporizers/
**7/20/2022**	vaporizer	https://www.vaporizerwizard.com/best-portable-vaporizers/

**7/20/2022**	vaporizer	https://www.smokecartel.com/collections/vaporizers
**7/20/2022**	vaporizer	https://www.vapor.com/
**7/20/2022**	vaporizer	https://www.vaporizerchief.com/vaporizers
**7/20/2022**	vaporizer	https://tvape.com/
**7/20/2022**	vaporizer	https://slickvapes.com/
**7/20/2022**	vape pen	https://vaping.com/starter-kits/vape-pens
**7/20/2022**	vape pen	https://vapingvibe.com/best-vape-pens/
**7/20/2022**	vape pen	https://www.hemper.co/blogs/news/a-guide-to-buying-your-first-vape-pen
**7/20/2022**	vape pen	https://discountvapepen.com/
**7/20/2022**	vape pen	https://slickvapes.com/collections/vape-pen
**7/20/2022**	vape pen	https://www.vaporfi.com/vape-pens/
**7/20/2022**	vape pen	https://www.vapor.com/collections/vape-pens
**7/20/2022**	electronic cigarettes	https://vaping360.com/best-beginner-e-cigs-vapes/
**7/20/2022**	electronic cigarettes	https://vapingdaily.com/best-electronic-cigarettes/
**7/20/2022**	electronic cigarettes	https://www.vaporfi.com/electronic-cigarettes/
**7/20/2022**	electronic cigarettes	https://epuffer.com/e-cigs/
**7/20/2022**	electronic cigarettes	https://shop.njoy.com/login.php
**7/20/2022**	electronic cigarettes	https://www.juul.com/
**7/20/2022**	electronic cigarettes	https://www.smokefreeonline.com/
**7/20/2022**	electronic cigarettes	https://www.iloveecigs.com/vape-shops-california
**7/20/2022**	electronic cigarettes	https://www.whitecloudelectroniccigarettes.com/
**7/20/2022**	electronic cigarettes	https://www.blu.com/en/US/e-cigs
**7/20/2022**	e-cigarettes	https://vaping360.com/best-beginner-e-cigs-vapes/
**7/20/2022**	e-cigarettes	https://vapingdaily.com/best-electronic-cigarettes/
**7/20/2022**	e-cigarettes	https://vapingvibe.com/best-nicotine-free-vape-products/
**7/20/2022**	e-cigarettes	https://vaping.com/
**7/20/2022**	e-cigarettes	https://www.vaporfi.com/electronic-cigarettes/
**7/20/2022**	e-cigarettes	https://epuffer.com/e-cigs/
**7/20/2022**	e-cigarettes	https://www.juul.com/
**7/20/2022**	e-cigarettes	https://shop.njoy.com/login.php
**7/20/2022**	e-cigarettes	https://www.myvaporstore.com/

**7/20/2022**	e-cigarettes	https://www.eleafworld.com/isolo-air-2/
**7/21/2022**	e-cigs	https://vaping360.com/best-beginner-e-cigs-vapes/
**7/21/2022**	e-cigs	https://vapingdaily.com/best-electronic-cigarettes/
**7/21/2022**	e-cigs	https://www.vaporfi.com/electronic-cigarettes/
**7/21/2022**	e-cigs	https://vaping.com/
**7/21/2022**	e-cigs	https://www.juul.com/
**7/21/2022**	e-cigs	https://www.blu.com/en/US/e-cigs
**7/21/2022**	e-cigs	https://epuffer.com/e-cigs/
**7/21/2022**	e-cigs	https://njoy.com/us/
**7/21/2022**	e-cigs	https://www.myvaporstore.com/
**7/21/2022**	e-cigs	https://www.iloveecigs.com/vape-shops-california
**7/21/2022**	hookah pens	https://www.everydayhookah.com/
**7/21/2022**	hookah pens	https://www.smoking-hookah.com/e-hookahs
**7/21/2022**	hookah pens	https://www.hookahshisha.org/portable-hookah-stick-c-39/
**7/21/2022**	hookah pens	https://www.texashookah.com/ehookahs.html
**7/21/2022**	hookah pens	https://www.amazon.com/shisha-pen/s?k=shisha+pen
**7/21/2022**	hookah pens	https://www.etsy.com/market/hookah_pen
**7/21/2022**	hookah pens	https://www.blakksmoke.com/product-page/blakk-smoke-on-the-go-hookah-pen-bundle
**7/21/2022**	hookah pens	https://www.dhgate.com/wholesale/hookah+pens.html
**7/21/2022**	e-cigars	https://epuffer.com/e-cigars/
**7/21/2022**	e-cigars	https://www.cuvanaecigar.com/
**7/21/2022**	e-cigars	https://mipod.com/products/ecigar
**7/21/2022**	e-cigars	https://www.apolloecigs.com/en/apollo-electronic-cigar-e-cigar
**7/21/2022**	e-cigars	https://vaping360.com/best-beginner-e-cigs-vapes/
**7/21/2022**	e-cigars	https://vapingdaily.com/best-electronic-cigarettes/
**7/21/2022**	e-cigars	https://www.jrcigars.com/vapes-and-e-cigs/
**7/21/2022**	e-cigars	https://www.aristocigars.com/
**7/21/2022**	e-cigars	https://vapingdaily.com/best-electronic-cigarettes/electronic-cigar/
**7/21/2022**	e-pipes	https://epuffer.com/e-pipe-vape-pipe/
**7/21/2022**	e-pipes	https://ecigarettereviewed.com/vape-pipes-e-cigars/
**7/21/2022**	e-pipes	https://www.vapeyaya.com/Premium-E-cig-E-Pipe

**7/21/2022**	e-pipes	https://www.smokstore.com/ECigKits/Pipe-Style-Kits
**7/21/2022**	e-pipes	https://vaping360.com/learn/e-pipes/
**7/21/2022**	e-pipes	http://vapegrl.com/e-pipes/
**7/21/2022**	e-pipes	https://www.etsy.com/market/e_pipe
**7/21/2022**	e-pipes	https://www.made-in-china.com/price/e-cigarette-e-pipe-618-price.html
**7/21/2022**	e-pipes	https://www.dhgate.com/wholesale/e+pipes+mods.html
**7/21/2022**	e-liquid	https://www.eightvape.com/collections/e-liquid
**7/21/2022**	e-liquid	https://www.directvapor.com/e-liquids/
**7/21/2022**	e-liquid	https://www.ecigarettedirect.co.uk/ashtray-blog/2020/01/e-liquid-buying-guide.html
**7/21/2022**	e-liquid	https://versedvaper.com/best-online-vape-stores/
**7/21/2022**	e-liquid	https://www.eightvape.com/?sscid=71k6_kb88t
**7/21/2022**	e-liquid	https://www.centralvapors.com/?sscid=71k6_kb89l
**7/21/2022**	e-liquid	https://myvpro.com/?sscid=71k6_kb89z
**7/21/2022**	e-liquid	https://vapordna.com/?sscid=71k6_kb8au
**7/21/2022**	e-liquid	https://www.directvapor.com/?sscid=71k6_kb8be
**7/21/2022**	e-liquid	https://www.ejuice.deals/?sscid=71k6_kb8cn&utm_source=ShareASale
**7/21/2022**	e-liquid	https://www.ejuices.com/?utm_source=27001&utm_content=1077519_1700915&sscid=71k6_kb8e2
**7/21/2022**	e-liquid	https://vapejuicedepot.com/?sscid=71k6_kb8ew&
**7/21/2022**	e-liquid	https://mipod.com/?sscid=71k6_kb8g9&utm_source=ShareASale&utm_medium=Affiliate&utm_campaign=1700915
**7/21/2022**	e-liquid	https://www.eliquid.com/
**7/21/2022**	e-liquid	https://www.ejuice.deals/
**7/21/2022**	e-liquid	https://www.ejuicestore.com/
**7/21/2022**	e-liquid	https://www.zamplebox.com/e-juice
**7/21/2022**	vape juice	https://www.eightvape.com/collections/e-liquid
**7/21/2022**	vape juice	https://www.ejuice.deals/
**7/21/2022**	vape juice	https://www.ejuicestore.com/
**7/21/2022**	vape juice	https://www.directvapor.com/e-liquids/
**7/21/2022**	vape juice	https://versedvaper.com/best-online-vape-stores/
**7/21/2022**	vape juice	https://www.eightvape.com/?sscid=71k6_kb88t

**7/21/2022**	vape juice	https://www.centralvapors.com/?sscid=71k6_kb89l
**7/21/2022**	vape juice	https://myvpro.com/?sscid=71k6_kb89z
**7/21/2022**	vape juice	https://vapordna.com/?sscid=71k6_kb8au
**7/21/2022**	vape juice	https://www.directvapor.com/?sscid=71k6_kb8be
**7/21/2022**	vape juice	https://www.ejuice.deals/?sscid=71k6_kb8cn&utm_source=ShareASale
**7/21/2022**	vape juice	https://www.ejuices.com/?utm_source=27001&utm_content=1077519_1700915&sscid=71k6_kb8e2
**7/21/2022**	vape juice	https://vapejuicedepot.com/?sscid=71k6_kb8ew&
**7/21/2022**	vape juice	https://mipod.com/?sscid=71k6_kb8g9&utm_source=ShareASale&utm_medium=Affiliate&utm_campaign=1700915
**7/21/2022**	vape juice	https://ejuicedirect.com/
**7/21/2022**	vape juice	https://westcoastvapesupply.com/collections/vape-juice
**7/21/2022**	vape juice	https://www.zamplebox.com/e-juice
**7/21/2022**	vape juice	https://www.eliquid-depot.com/
**7/21/2022**	Dab pens	https://www.smokecartel.com/collections/dab-pens-wax-vaporizers
**7/21/2022**	Dab pens	https://slickvapes.com/collections/wax-dab-pen
**7/21/2022**	Dab pens	https://tvape.com/blog/best-wax-pens/
**7/21/2022**	Dab pens	https://vapingdaily.com/cheap-dab-wax-pens/
**7/21/2022**	Dab pens	https://thevape.guide/best-wax-dab-vape-pen/
**7/21/2022**	Dab pens	https://everythingfor420.com/collections/dab-pens
**7/21/2022**	Dab pens	https://mindvapes.com/collections/dab-vape-pens
**7/21/2022**	Dab pens	https://www.vape4ever.com/dab-pens-wax-vape-pens_c261.html
**7/21/2022**	Dab pens	https://www.drdabber.com/
**7/21/2022**	Dab pens	https://www.grasscity.com/vaporizers/dab-wax-pens
**7/21/2022**	Disposables	https://www.eightvape.com/collections/disposable-vape-pen
**7/21/2022**	Disposables	https://vapordna.com/collections/disposable-vaporizers
**7/21/2022**	Disposables	https://www.electrictobacconist.com/disposable-e-cigs-c4
**7/21/2022**	Disposables	https://mipod.com/collections/disposable-vape
**7/21/2022**	Disposables	https://vaporempire.com/disposables/
**7/21/2022**	Disposables	https://vaping.com/starter-kits/disposable-vapes
**7/21/2022**	Disposables	https://www.huffandpuffers.com/collections/disposable-salt-nicotine-devices

**7/21/2022**	Disposables	https://versedvaper.com/best-disposable-e-cigs/
**7/21/2022**	Disposables	https://vapingdaily.com/best-electronic-cigarettes/disposable-e-cigs/
**7/21/2022**	Disposables	https://thevaporshoppeusa.com/collections/disposables
**7/21/2022**	Mods	https://vapordna.com/collections/vape-devices
**7/21/2022**	Mods	https://www.elementvape.com/
**7/21/2022**	Mods	https://vaping360.com/best-vape-mods/
**7/21/2022**	Mods	https://vapingdaily.com/best-vape-mods/
**7/21/2022**	Mods	https://www.eightvape.com/collections/mods
**7/21/2022**	Mods	https://giantvapes.com/collections/all-mods-kits
**7/21/2022**	Mods	https://www.directvapor.com/
**7/21/2022**	Mods	https://www.vaporfi.com/vape-mods/
**7/21/2022**	Pods	https://buypodsnow.com/
**7/21/2022**	Pods	https://www.juul.com/shop/pods
**7/21/2022**	Pods	https://www.podsoutlet.com/
**7/21/2022**	Pods	https://www.vaporfi.com/vape-accessories/pod-cartridges/
**7/21/2022**	Pods	https://www.podvapes.com/
**7/21/2022**	closed pod system	https://buypodsnow.com/product-category/pod-systems/closed-pod-system/
**7/21/2022**	closed pod system	https://thenicsalt.com/collections/pod-systems
**7/21/2022**	closed pod system	https://www.ecigclick.co.uk/best-pod-mods-for-vaping/
**7/21/2022**	closed pod system	https://www.vapedinnerlady.com/blogs/vape-dinner-lady-blog/finding-the-best-refillable-pod-system-open-vs-closed-pod-systems-1
**7/21/2022**	closed pod system	https://www.kurevapes.com/collections/closed-end-pod-devices
**7/21/2022**	closed pod system	https://jeancloudvape.com/product-category/equipments/pod-2/closed-pod-system/
**7/21/2022**	closed pod system	https://vapingbear.com/collections/closed-pod-system
**7/21/2022**	closed pod system	https://www.180smoke.ca/closed-pod-vape-kits
**7/21/2022**	closed pod system	https://www.electrictobacconist.com/pod-mods-c51
**7/21/2022**	nic sticc	http://m.nicstick.com/
**7/21/2022**	nic sticc	https://www.electrictobacconist.com/disposable-e-cigs-c4
**7/21/2022**	nic sticc	https://ziipstick.com/
**7/21/2022**	nic sticc	https://www.elementvape.com/horizon-magico-pod-stick-kit
**7/21/2022**	nic sticc	https://www.vapelargest.com/magico-salt-nic-stick-kit

**7/21/2022**	nic sticc	https://bidivapor.com/login/
**8/3/2022**	nic sticc	https://www.budgetvapors.com/horizon-magico-nic-salt-stick-kit/
**8/3/2022**	nic sticc	https://vaping.com/horizontech-magico-nic-salt-stick-kit
**8/3/2022**	e-juice	https://www.eightvape.com/collections/e-liquid
**8/3/2022**	e-juice	https://ejuice.deals/
**8/3/2022**	e-juice	https://www.ejuicestore.com/
**8/3/2022**	e-juice	https://www.directvapor.com/e-liquids/
**8/3/2022**	e-juice	https://ejuicedirect.com/
**8/3/2022**	e-juice	https://westcoastvapesupply.com/collections/vape-juice
**8/3/2022**	e-juice	https://www.eliquid-depot.com/
**8/3/2022**	e-juice	https://www.ejuiceconnect.com/
**8/3/2022**	e-juice	https://www.elementvape.com/
**8/3/2022**	refillable devices	https://versedvaper.com/best-pod-vapes/
**8/3/2022**	refillable devices	https://ecigarettereviewed.com/best-pod-vapes/
**8/3/2022**	refillable devices	https://www.ecigclick.co.uk/best-pod-mods-for-vaping/
**8/3/2022**	refillable devices	https://cityofvape.com/refillable-pod-system-kits/
**8/3/2022**	refillable devices	https://vapordna.com/collections/open-vape-pod-systems
**8/3/2022**	refillable devices	https://vaping.com/starter-kits/pod-systems
**8/3/2022**	refillable devices	https://www.centralvapors.com/vape-pod-systems/
**8/3/2022**	refillable devices	https://www.electrictobacconist.com/pod-mods-c51
**8/3/2022**	refillable devices	https://vaping360.com/best-vape-mods/pod-vapes/
**8/3/2022**	open system	https://hazetownvapes.com/collections/open-pod-systems
**8/3/2022**	open system	https://vapordna.com/collections/open-vape-pod-systems
**8/3/2022**	open system	https://ridervs.com/collections/open-systems
**8/3/2022**	open system	https://the-kocky-dog.myshopify.com/collections/open-pod-systems
**8/3/2022**	open system	https://breazy.com/collections/pod-mods
**8/3/2022**	open system	https://www.elementvape.com/pod-systems
**8/3/2022**	open system	https://versedvaper.com/best-pod-vapes/
**8/3/2022**	open system	https://vaping360.com/best-vape-mods/pod-vapes/
**8/3/2022**	dank vapes	https://dankvapesonline.com/
**8/3/2022**	dank vapes	https://thedankvape.com/

**8/3/2022**	dank vapes	https://dankvape.org/
**8/3/2022**	dank vapes	https://dankvapesofficial.org/
**8/3/2022**	dank vapes	https://greenhousedispensary.store/product-category/vape-pens-cartridges/dank-vapes/
**8/3/2022**	dank vapes	https://dankvapetech.com/
**8/3/2022**	dank vapes	https://dankvapesonlinedisp.shop/
**8/3/2022**	dank vapes	https://www.buymarijuanastrains.com/dank-vapes.php
**8/3/2022**	dank vapes	https://420delivery.online/product/buy-dank-vapes-online/
**8/3/2022**	refillable system	https://cityofvape.com/refillable-pod-system-kits/
**8/3/2022**	refillable system	https://vapordna.com/collections/open-vape-pod-systems
**8/3/2022**	refillable system	https://vaping.com/starter-kits/pod-systems
**8/3/2022**	refillable system	https://www.centralvapors.com/vape-pod-systems/
**8/3/2022**	refillable system	https://www.vapeloft.com/category/vape-starter-kits/pod-vape/
**8/3/2022**	refillable system	https://vapecrypto.com/product-category/vape-pods/refillable-pod-systems/
**8/3/2022**	refillable system	https://saltnic.com/pod-systems/refillable-starter-kits.html
**8/3/2022**	refillable system	https://www.electrictobacconist.com/pod-mods-c51
**8/3/2022**	puff bars	https://puffbar.com/
**8/3/2022**	puff bars	https://puffecig.com/puff-bar-disposable-device/
**8/3/2022**	puff bars	https://www.eliquidstop.com/products/puff-bar-disposable-device
**8/3/2022**	puff bars	https://ezpuff.com/puff-bar-plus-all-flavors/
**8/3/2022**	puff bars	https://vaporboss.com/products/puff-bar-disposable-vape
**8/3/2022**	puff bars	https://www.fatpuffwholesale.com/where-to-get-bulk-puff-bars-cheap/
**8/3/2022**	puff bars	https://www.puffbarstudio.com/
**8/3/2022**	puff bars	https://www.alternativepods.com/puff-bar-plus-disposable/
**8/3/2022**	stig	https://stigpods.com/
**8/3/2022**	stig	https://ohmcityvapes.com/products/stig-pods-ultra-portable-vape-pod-system-or-device
**8/3/2022**	stig	https://www.pricepointny.com/collections/stig-i-disposable-pod-device
**8/3/2022**	stig	https://ashvapesmoke.com/products/stig-disposable-pod-device
**8/3/2022**	stig	https://thesmokeplug.com/products/vgod-stig-disposable-vape-pod-device-1pc
**8/3/2022**	stig	https://breazy.com/collections/stig
**8/3/2022**	stig	https://officialvgod.com/stig-disposable-pod-device
**8/3/2022**	stig	https://greencaviarclub.com/products/vgod-stig-disposable-vape-device-mighty-mint

**8/3/2022**	stig	https://saltnic.com/stig-disposable-pods.html
**8/3/2022**	cuvie	https://www.vapecentralgroup.com/products/hqd-root-500-puff-salt-nic-vape-device
**8/3/2022**	cuvie	https://ohmcityvapes.com/products/hqd-cuvie-plus-disposable-vape-device
**8/3/2022**	cuvie	https://www.hqdtechaus.com/shipping-policy/
**8/3/2022**	cuvie	https://hqdtechusa.com/
**8/3/2022**	cuvie	https://www.smokersworldhw.com/products/hqd-cuvie-air-disposable-vape-1-pack
**8/3/2022**	cuvie	https://suorinvape.com/collections/disposable-pod-systems/products/hqd-cuvie-ultimate-disposable-vape-5000-puffs?variant=42812627321059
**8/3/2022**	cuvie	https://www.vapor4all.com/products/hqd-cuvie-plus-vape
**8/3/2022**	cuvie	https://thesmokeplug.com/collections/hqd

© 2026 Apollonio D.E. et al.
